# Monitoring the Degradation of Island Permafrost Using Time-Series InSAR Technique: A Case Study of Heihe, China

**DOI:** 10.3390/s19061364

**Published:** 2019-03-19

**Authors:** Sai Wang, Bing Xu, Wei Shan, Jiancun Shi, Zhiwei Li, Guangcai Feng

**Affiliations:** 1 School of Geosciences and Info-Physics, Central South University, Changsha 410083, China; wangsai@csu.edu.cn (S.W.); jiancun.shi@csu.edu.cn (J.S.); zwli@csu.edu.cn (Z.L.); fredgps@csu.edu.cn (G.F.); 2 Engineering Consulting and Design Institute, Northeast Forestry University, Harbin 150040, China; shanwei456@163.com

**Keywords:** island permafrost, deformation, time-series InSAR, global warming, geohazards

## Abstract

In the context of global warming, the air temperature of the Heihe basin in Northeast China has increased significantly, resulting in the degradation of the island permafrost. In this paper, we used an elaborated time-series Interferometric Synthetic Aperture Radar (InSAR) strategy to monitor the ground deformation in the Heihe area (Heilongjiang Province, China) and then analyzed the permafrost deformation characteristics from June 2007 to December 2010. The results showed that the region presented island permafrost surface deformation, and the deformation rate along the line of sight mainly varied from –70 to 70 mm/a. Based on the analysis of remote sensing and topological measurements, we found that the deformation area generally occurred at lower altitudes and on shady slopes, which is consistent with the distribution characteristics of permafrost islands. Additionally, the deformation of permafrost is highly correlated with the increase of annual minimum temperature, with an average correlation value of –0.80. The accelerated degradation of permafrost in the study area led to the settlement, threatening the infrastructure safety. Our results reveal accelerated degradation characteristics for the island permafrost under the background of rising air temperature, and provide a reference for future relevant research.

## 1. Introduction

Seasonal permafrost and permafrost are broadly distributed in China, covering 54% and 25% of the land area, respectively. The distribution area of all permafrost is about 1.59 × 10^6^ km^2^, ranking third in the world. The permafrost distributed in the northern part of Northeast China represents the second largest area of permafrost in China [1]. In recent years, global warming has caused a rise in air temperatures and increasing precipitation, which have accelerated the degradation of permafrost. Due to the widespread degeneration of permafrost, forests are dramatically transforming into swamps, further breaking the ecosystem equilibrium. This situation should receive attention [2]. As a sensitive indicator of climate change, the change in permafrost has attracted special attention around the world.

Northeast China is the southern margin of the Eurasian permafrost region. The permafrost of these low-elevation islands is more sensitive to changes in climate and environmental, and has shown obvious signs of degradation in recent years. The surface deformation caused by island permafrost degradation is localized, which is different from the surface deformation (usually the overall uplift or subsidence) caused by permafrost degradation. Additionally, the degradation of island permafrost has a great impact on the local environment, leading to phenomena such as landslides [3], solifluction, and partial expressway collapse and destruction. With the increase of construction projects, understanding the distribution of surface deformation in the island permafrost area is the key to disaster warning and prevention. However, compared with the permafrost in the Qinghai–Tibet plateau, the permafrost monitoring research in this region is insufficiently studied. More attention needs to be paid to the permafrost degradation in the future.

Due to its wide spatial coverage, Interferometric Synthetic Aperture Radar (InSAR), as an all-weather monitoring method with competitive accuracy, has been widely used to obtain ground deformation measurements. The traditional differential InSAR technique is inevitably affected by spatiotemporal decorrelation, atmospheric delay, and random noise, which limits the measurement accuracy to some extent [4]. To overcome these limitations, time-series InSAR techniques such as persistent scatterer interferometry (PS-InSAR) [5] and small-baseline subset interferometry (SBAS-InSAR) have been proposed [6]. These time-series InSAR techniques can monitor small deformation over a long period, are suitable for surface deformation inversion in the study area, and have been successfully applied in many cases. The InSAR technique has been widely used for monitoring surface deformation caused by earthquakes [7], underground mining [8], landslides [9], oil and gas extraction [10], groundwater extraction [11], and thawing and freezing of permafrost [12,13]. 

In this study, we focus on the degradation monitoring of island permafrost around the expressway from Bei’an to Heihe in Heilongjiang Province, China. First, a set of 20 scenes L-band ALOS/PALSAR stripmap images acquired from June 2006 to December 2010 were processed with an elaborated time-series InSAR strategy to obtain deformation of the study area. Subsequently, the deformation of island permafrost was analyzed in combination with elevation, slope direction, and temperature. Moreover, we analyzed the correlation between the deformation of island permafrost and elevated temperature. Finally, we further analyzed the potential geological hazards associated with island permafrost degradation.

## 2. Study Area

**Figure 1:**
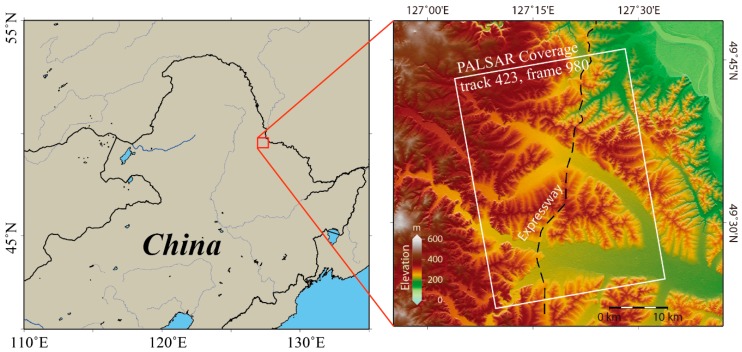
The study area and its Digital Elevation Model (DEM). The red rectangle on the left outlines the spatial coverage of the study area, and the right inset shows the DEM of the study area, with the bold black dashed line denoting the expressway from Bei’an to Heihe and the white rectangle denoting the coverage of the ALOS/PALSAR data used in this study.

The study area is located in the Sunwu–Aihui area, Heihe City, in Northern Heilongjiang Province, China (see Figure 1). The plains of the study area have low mountainous terrain, with an elevation of between 140 and 425 m. The elevation of the study area gradually decreases from the northwest to the southeast. 

The central part of the image is intermountain depressions and valley terraces, distributed in marshes, cultivated land, and other land types, while the south part is mainly cultivated land. The study area is located in the transitional region between the middle-temperate zone and the cool-temperate zone, and is alternately affected by low pressure and high pressure from the surrounding area, as well as by the monsoon [14].

The climate in the study area has strong seasonal characteristics, with cold, dry, and long winters, and hot, humid, and short summers. The annual average precipitation is about 540 mm, although can sometimes reach up to 800 mm. The precipitation mainly occurs in the form of rainfall from July to September, accounting for 61% to 67% of the total annual precipitation [15]. The annual average temperature is about −2 to 2 °C. Meteorological data indicate that the air temperature has exhibited a significant variation [16]. China’s air temperature increase rate of 0.022 °C/a in the past 50 years is higher than the global or Northern Hemisphere average temperature increase rate [17]. The northeast region of China, with an air temperature increase rate of 0.035 °C/a, is one of the areas with the fastest temperature rise in China.

**Figure 2:**
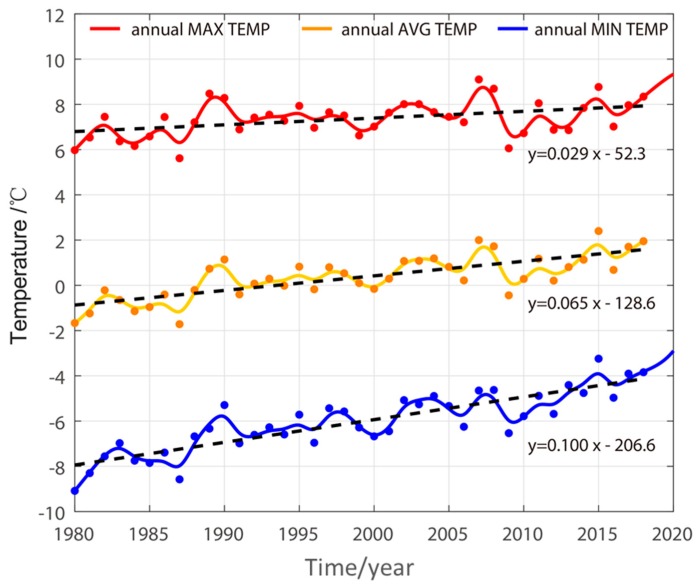
The annual air temperature of Sunwu County, Heilongjiang Province, China, from 1980 to 2018.

The rate of air temperature warming in the Sun Wu area is the highest in Northeast China [16]. According to the temperature data of the Sunwu County Meteorological Station, from 1980 to 2018, the annual average temperature, the annual maximum temperature, and the annual average minimum temperature were plotted in Figure 2. The temperature change rate during the 39 years from 1980 to 2018 was obtained by linear fitting. The annual average minimum temperature increased at a significant rate of 0.1 °C /a, which is the largest and about 3.3 times the annual average maximum air temperature increases in Northeast China.

The spatial distribution of island permafrost in the study area is highly variable and discrete. The temperature, thickness, and thermal stability of permafrost, and the sensitivity of island permafrost to climatic and environmental changes have obvious local spatiotemporal differences [18]. In the context of climate change, a series of geological disasters, such as landslides, soil erosion, and debris flows are prone to occur, and the severe soil impoverishment caused by island-type permafrost degradation may occur in some areas.

The Bei’an to Heihe Expressway, which was built between 1997 and 2000, was unstable due to the melting of permafrost in 1999. Subsequently, the road was repaved to a ridge with stable geological conditions. Since 1994, the area of cultivated land in the study area has increased, and the forest area has decreased dramatically. The surface temperature and its amplitude has increased, the seasonal melting depth has increased, and the total area of island permafrost has gradually decreased. Since 2000, the permafrost in the study area has been extensively degraded.

In general, the degraded permafrost in our study area is highly sensitive under the described climatic conditions [19]. Any sustained warming or human activity can accelerate permafrost degradation, further destabilizing slopes and increasing the damage associated with permafrost. The InSAR monitoring results presented in this paper are of great significance for environmental assessment and regional infrastructure security in the study area.

## 3. Data Processing and Results

In our study, a total of 20 L-band ALOS/PALASAR stripmap images over the Heihe area were used to retrieve the ground deformation caused by permafrost degeneration. The dataset was acquired along the ALOS satellite track 423 from June 2007 to December 2010 with Fine Beam Single (FBS) polarization or Fine Beam Double (FBD) polarization mode. The incidence angle of the image center point was about 38.75°. Table 1 details the information of the dataset used. The data coverage is shown in Figure 1. Due to the dense vegetation cover in our region of interest and the periodical change of water content in the permafrost active layer, the application of InSAR is mainly limited by temporal decorrelation [20]. Nevertheless, the long wavelength (λ ≈ 23.6 cm) of the L-band is less affected by this limitation and maintains good coherence in the study area [20,21].

**Figure 3:**
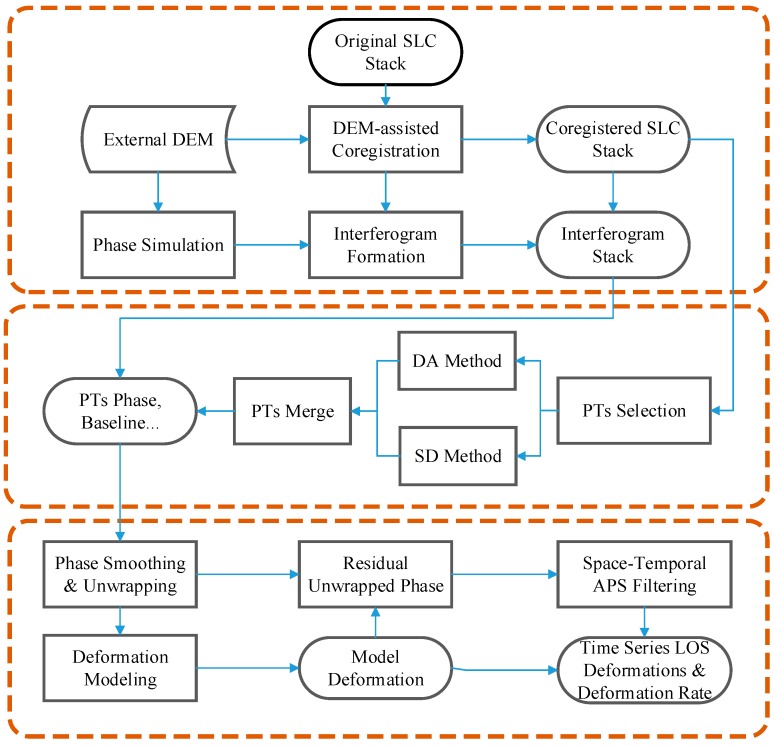
The elaborated data processing strategy.

In order to obtain highly coherent interferometric results, an elaborated data processing strategy combining the SBAS-InSAR and PS-InSAR methods is used to process a long dataset. Figure 3 shows an elaborated data processing strategy for retrieving the deformation time-series and the corresponding mean deformation velocity. The processing strategy consists of three major parts, namely pre-processing, point targets selection, and deformation time-series retrieval.

**Figure 4:**
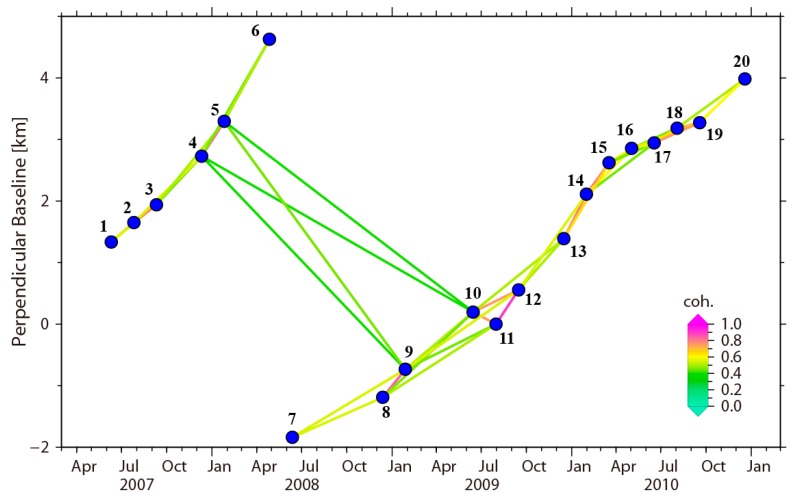
The network of the selected interferometric pairs. Blue dots denote a SAR image acquisition. Lines between blue dots are interferometric pairs, the colors of which represent the average coherence (coh.) of the corresponding interferogram.

Data processing starts with a stack of focus Single Look Complex (SLC) images. Firstly, the GAMMA software (v20180704) [22] was used to import the dataset. Then, all SLC images were co-registered in GAMMA software. The image acquired on 31 July 2009 (No. 11, see Table 1) was chosen as the reference image for SLC co-registration by minimizing the decorrelation. To reduce the topographic effect in image range offset, the SLC stack was co-registered with the geometrical SAR image co-registration method assisted by 1-arc-second (spatial resolution is about 30 m × 30 m) SRTM Digital Elevation Model (DEM). We only considered interferometric pairs with short perpendicular baseline and temporal baseline, i.e., 1.5 km, which is significantly smaller than the critical baseline [20]. Figure 4 shows the network of selected interferometric pairs. Since the ALOS orbit was adjusted in mid-2008 [23], the long time-series images were divided into two subsets due to the large separation of baseline (i.e., 6–7). To connect the two subsets, several pairs with a relatively long baseline, namely 4–9, 4–10, 5–9, and 5–10, were selected manually. The coherences of these pairs remained at an acceptable level (coherence ≥ 0.4).

Subsequently, the original interferograms were generated by conjugate multiplication between the co-registered master SLC image and slave SLC image. Thus, a DEM was interpolated from 1-arc-second SRTM DEM, which had the same regular grid spacing as the reference SLC image. We utilized the DEM to simulate and remove the topographic phase contribution using the GAMMA software during the interferogram generation process. Then, we geocoded InSAR results from range-Doppler coordinates into map geometry corresponding to the WGS84 coordinate system using the GAMMA software.

The selection of highly coherent point targets (PTs) is one of the key steps for our processing strategy and analysis. Currently, spatial coherence is usually used as a criterion for PTs selection. However, the coherence would be overestimated in a lowly coherent area [24], as in the case of our study area. As a consequence, there would be many unreliable PTs selected. To select out reliable PTs, the Spectral Diversity (SD) [25] (implemented in GAMMA software) and Amplitude Dispersion (DA) method [26] derived from PS-InSAR [5] were used to select PTs with the coregistered SLC images, respectively. The spectral correlation minimum threshold was set to 0.30 and the DA threshold was set to 0.25. The final PTs list was the union of the PTs selected by the SD method and the PTs selected by the DA method. The interferometric phases and baseline information for each PT were extracted from the data grid. 

**Figure 5:**
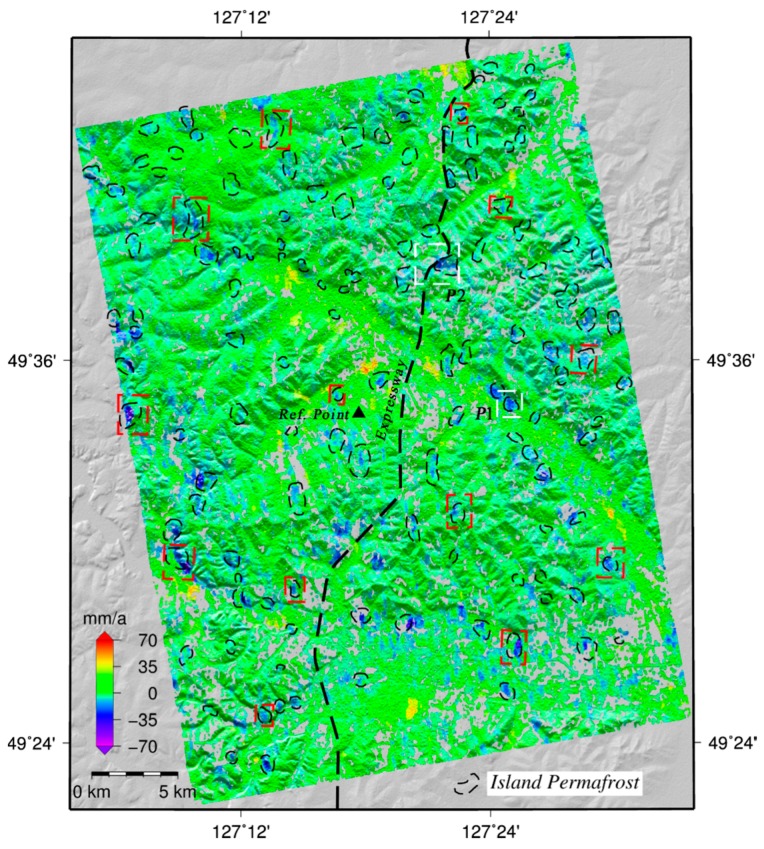
Average annual deformation rate along the line of sight direction. The background is DEM shaded relief, and the black dashed polygons denote the island permafrost areas, which were derived from Landsat images in our previous work [28]. The black triangle denotes the reference point, the bold black dashed line denotes the expressway from Bei’an to Heihe, and the white and red rectangles denote selected areas for further analysis in this study.

In order to alleviate the phase random noise, the interferometric phase was smoothed by a low-pass adaptive phase filter prior to phase unwrapping. The reference point was set to a stable area near the image center (see the black triangle in Figure 5). The smoothed phases of PTs for each interferometric pair were unwrapped by a network programming-based phase unwrapping method [27]. Subsequently, the SBAS-InSAR method [6] was implemented to obtain the deformation time-series and the corresponding mean deformation velocity in the study area. A triangular filter was used for temporal and spatial atmospheric screen estimation [5,26].

Finally, we obtained the deformation rate and the deformation time-series along the line of sight from 2006 to 2010 (refer to 10 June 2006). As shown in Figure 5, the result was plotted on a DEM shade relief. The deformation rate ranged from –70 to 70 mm/a. Approximately 530,000 PTs were obtained in the experiment, most of which were nearly stable or showed slight deformation (ranging from −35 to 35 mm/a). However, there were some island-shaped deformation regions having a significant deformation rate. In order to show more details of the deformation, the island permafrost area was superimposed on the deformation rate figure [14]. It can be seen that most of the island permafrost area underwent significant deformation, substantially greater than 35 mm/a. These results indicate that the island permafrost was degrading. In addition, we shall note that there were several sites with positive deformation rate, meaning that the ground settlement moved towards the satellite. However, the positive deformation only occurred on low altitude or slope facing to satellite, see Figure 5. Such positive deformation is very interesting. The high-altitude slope deformed, and the material migrated to lower areas, leading to possible uplift. Besides, when a slope facing to satellite, the slope settlement would also cause positive deformation rate. For the sake for simplicity, we only focused on the negative deformation rate in this paper.

## 4. Results, Analysis, and Discussion

### 4.1. Deformation Statistics with Topographic Factors

It can be seen from Figure 5 that the deformation in the study area mainly occurred in the low-altitude valleys or flat areas, while there was almost no deformation in the mountainous areas. To further explore the relationship between ground deformation and elevation, we conducted a statistical analysis of the altitude and the deformation rate. Firstly, the elevation of the study area was divided into nine groups with an equal interval of 30 m. Then, we counted the number of PTs with significant deformation (i.e., a deformation rate >20 mm/a or < −20 mm/a) in each elevation group and calculated corresponding proportion. 

**Figure 6:**
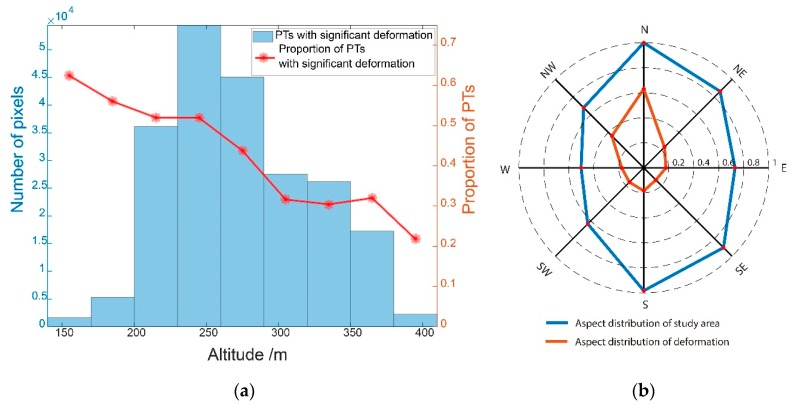
(**a**) significant deformation distribution at different altitudes. (**b**) aspect distribution of study area and deformation.

The statistical result is shown in Figure 6a. It can be seen that the proportion of PTs decreases with the altitude rises. Most of the significant deformation was located at lower altitudes of 210–300 m, and that the altitude of 250 m had the highest possibility to deform. The results imply that the island permafrost at these altitudes was more possible to deform than at other elevations. Our results are in accordance with previous work [28], which found that the island permafrost was distributed in low-lying areas in Heilongjiang Province.

The solar irradiance is greatest on the southern slope, followed by the southeast slope, southwest slope, east slope, and west slope. The solar irradiance on the northeast slope, northwest slope, and north slope are the smallest. Figure 6b shows the slope distribution statistics of the study area and the slope distribution statistics of the PTs with significant deformation, obtained by superposition analysis of the deformation area and the slope direction obtained by 1 arc-second SRTM DEM. We found that the deformation area was distributed in shadow with less solar radiation, including the north slope, the northwest slope, and the northeast slope. This is consistent with the slope distribution of permafrost in study area.

### 4.2. Correlation Analysis with Air Temperature

The island-shaped and sparse island-like permafrost in the study area is widely distributed, and is the transition zone between permafrost and seasonal permafrost. Permafrost is extremely sensitive to surface heat exchange conditions [29]. According to the temperature monitoring data of Sunwu County Meteorological Station, since 1981, the temperature in the study area had shown a significant rising trend. The average annual temperature of Sunwu County from 1981 to 2018 is shown in Figure 3. Regarding the trend of rising temperature, it can be found that the annual average minimum temperature growth in the region is the fastest, with a growth rate of 0.1 °C/a. The increase of the annual average minimum temperature in the study area is greater than the increase of the annual average maximum temperature, indicating that the main reason for the increase in temperature is not the increase in direct radiation but the decrease of soil heat demand and the increase of soil radiation to the atmosphere [16].

**Figure 7:**
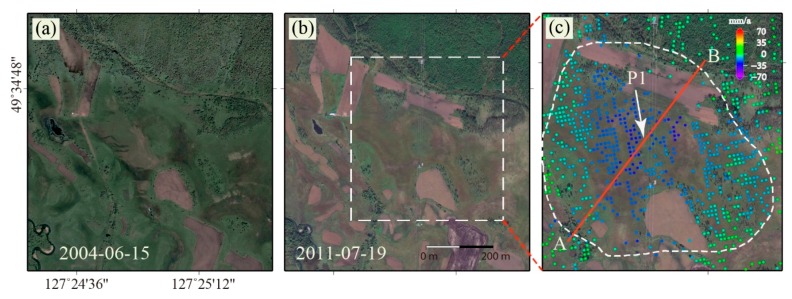
Optical images of selected area P1 and its deformation. (**a**) image acquired on 15 June 2004; (**b**) image acquired on 19 July 2011; (**c**) magnification of the white dashed rectangle in (c) with the deformation rate overlap; the white dashed polygon denotes the island permafrost area.

In order to show further details of the experimental results, several island permafrost deformation areas were selected for analysis. Selected area P1 (see the white rectangle in Figure 5) is located on the beach of a river valley, as shown in Figure 7, where the water content in soil is high, the altitude is low, and the terrain is flat. Compared with the optical images acquired in 2004 and 2011, it can be seen that the land surface features had changed. 

**Figure 8:**
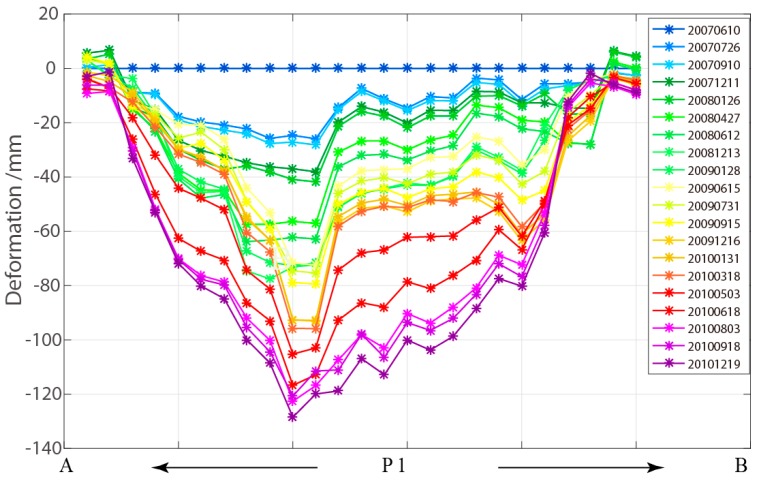
Profile of deformation time-series along the red line in Figure 7c for the selected area P1.

According to the land use dynamic change of Sunwu County from 1996 to 2010, the increase rate of arable land and garden land was about 118% and 727%, and the forest land decreased by about 22% [30]. The ever forest vegetation was replaced by reclaimed farmland, development of urban construction implying that the human activities had aggravated the degradation process of permafrost in this region. Due to the interference of human activities, the island permafrost (see Figure 7c) underwent continued degradation, causing ground deformation. The deformation profile of area P1 is shown in Figure 8. The result shows obvious ground deformation, indicating that island permafrost was undergoing degradation. According to the comparison of the optical images acquired in the same season (see Figure 7a,b), we can see that the ever-vegetated areas have been reclaimed for agricultural use. This change leads us to believe that the degradation of island permafrost will be accelerated by human activities.

**Figure 9:**
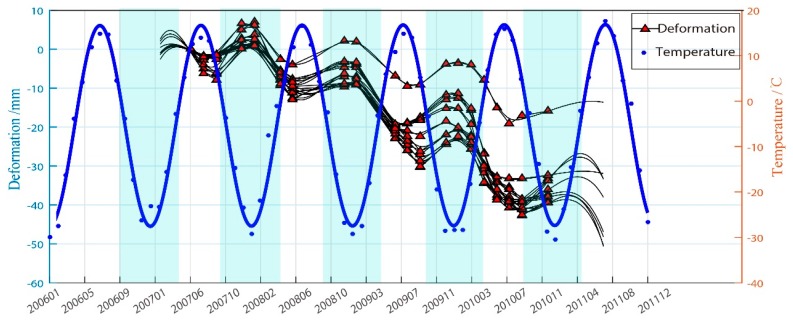
The deformation time-series for area P1 and the temperature.

Furthermore, in order to show the relationship between the air temperature and ground deformations, we plotted the deformations of 13 selected areas (see red dashed rectangles in Figure 5) and air temperature in Figure 9. In the warm season, from April to September, the temperature causes the soil to absorb more heat than it loses, especially in July and August, when the temperature rises dramatically. Consequently, the permafrost melting and surface subsidence peaked. The temperature started to drop around October, and the heat released from depth in the soil was greater than the heat absorbed from the atmosphere, which caused the water in the soil to freeze, resulting in frost heaving of the ground surface. 

**Figure 10:**
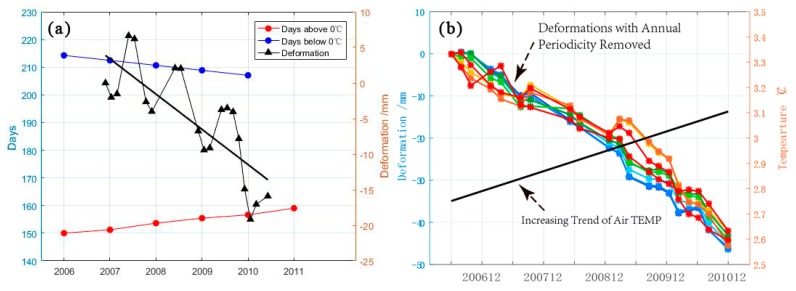
(**a**) the deformation of area P1 and the period of below/above 0 °C; (**b**) linear residual of deformation and fitting linear residual of air temperature.

The time to reach the maximum melting depth and the time to reach the maximum degree of frost heave will be slightly later than the time when the highest/lowest temperature peak appears, which is consistent with the general law of permafrost deformation. Furthermore, we calculated the deformation lag time to be about 25 days. The deformation fitting curves (see Figure 9) are composed of an annual periodic function and a linear function. Periodicity is due to seasonal variation. The linear subsidence is related to the degradation of permafrost. After the first winter (2007) in the study period, a freezing process was carried out, followed by an apparent settlement in the summer. In the next two years, the frost heave of permafrost in winter was lower than the deformation variable caused by melting settlement. The subsidence trend caused by permafrost degradation was obvious. It can also be clearly seen from the change of the positive and negative temperature duration of the annual average minimum temperature in Sunwu County during the superposition study period that the days with continuous negative temperature decreased and the days with continuous positive temperature increased. Based on the change of the annual air temperature, not only did the average minimum temperature rise, but the corresponding period below 0 °C also shortened in our study area (see Figure 10a). The change indirectly indicates that the permafrost melting period is prolonged and the corresponding frost heave period is shortened. Such climate change is likely to lead to the degradation of island permafrost in the study area. 

To further illustrate the correlation between air temperature change and deformation, we removed the annual periodicity from temperature change and deformation, respectively, and obtained the corresponding linear trend, as shown in Figure 10b. By analyzing the correlation between the deformation trend and the temperature increase trend, it was found that the correlation between the deformation trend and the temperature change varies from −0.78 to −0.85, with an average of −0.80. This indicates that air temperature increase is an important factor affecting island permafrost deformation.

### 4.3. Potential Geological Hazards

**Figure 11:**
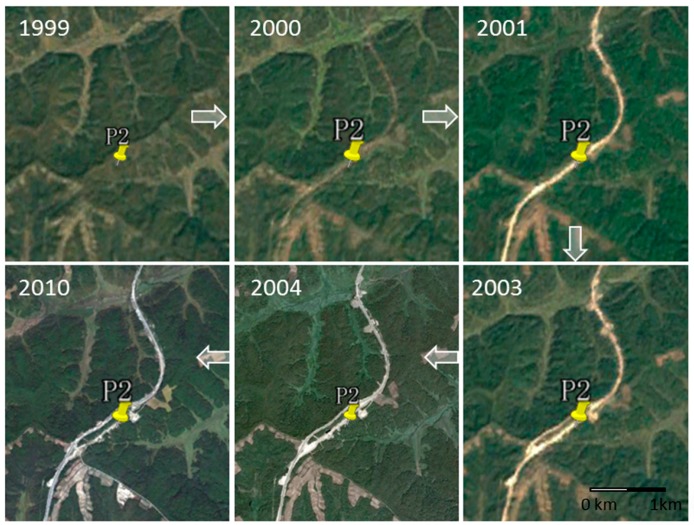
Historic changes of the Bei’an–Heihe expressway (Landsat images).

Under global climate warming conditions, the permafrost environment in the Heihe area is facing a great threat. The physical and mechanical properties of the original permafrost have changed greatly [31]. As an early project developed and constructed in the northern permafrost region, the Bei’an–Heihe expressway section is located in the permafrost degradation region. Being in a state of vulnerability, permafrost is very sensitive to human activities, such as engineering construction. Even weak thermal influence will lead to great changes that accelerate permafrost degradation. The changes related to the Bei’an–Heihe expressway, a selected area P2 (see the white rectangle in Figure 5), are shown in Figure 11. The expressway, which was designed and constructed from 1999 to 2000, was originally located on lower altitude slopes. However, the permafrost slope became unstable after construction [32], and subsequently a realignment of the expressway was carried out in 2003. 

**Figure 12:**
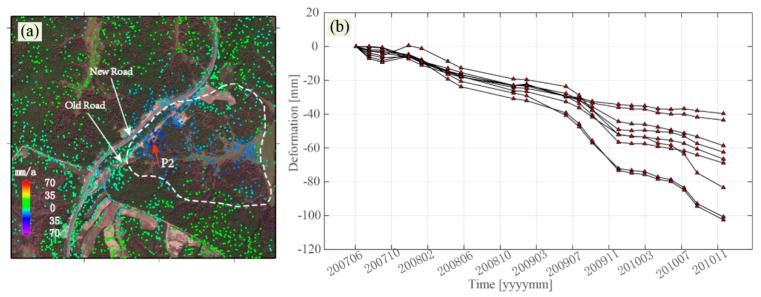
(**a**) deformation of the selected area P2. The white dashed polygon denotes the island permafrost. (**b**) the deformation time-series in the region indicated by the red arrow.

The InSAR monitoring results show that there was significant ground deformation caused by permafrost melting, as shown in Figure 12. It can be seen that the new road has been shifted to a higher altitude, which is out of the island permafrost area. However, there is still significant deformation threatening the expressway. The deformation time-series for the selected area P2 presents a stepped shape, and the overall trend is downward. It can be seen that this region experienced almost no thawing deformation in summer and permafrost frost heaving in winter. With the increase of air temperature, the water content in the soil increased after the permafrost degraded, which decreased the friction of the active layer. The soil was prone to deform with the factor of gravity on the slope (about 15°), and the deformation rate of the permafrost increased. This is a reflection of the rapid degradation of the island permafrost in the study area under the increasing air temperature.

It can also be seen from this case that mechanical properties such as stress and strain of permafrost are affected by thawing due to the influence of air temperature increase. Besides, farming and/or deforestation activities are also factors which accelerate the permafrost degradation. As a consequence, geological disasters such as landslides tend to occur. All of these factors will bring difficulties to local engineering investigation, construction design, quality control, and maintenance. InSAR, as a practical technique, can be applied to monitor and analyze the ground deformation of permafrost areas that are challenging for conducting field investigations.

## 5. Conclusions

In the context of global warming, the air temperature of Heihe, China, has increased significantly, resulting in the degradation of island permafrost. In this paper, a large-scale ground deformation survey was conducted in the Heihe area by using a time-series InSAR technique. The findings are concluded as follows:

(1) The time deformation of the Bei’an–Heihe area was obtained. From 2007 to 2010, the area had a deformation rate of −70 to 70 mm/a. The ground deformation caused by the thawing of permafrost was greater than that caused by frost heaving. The settlement characteristics of the permafrost in the study area were closely related to the altitude, being mainly distributed in low-altitude areas.
(2) The significant increase of temperature has intensified the development of warming and drying trends in the Heihe area. The island permafrost in the study area is sensitive to climate change. Based on a joint analysis with temperature, we found that the correlation between the deformation trend and temperature change varied from −0.78 to −0.85, with an average value of −0.80, indicating a significant correlation between degradation grade and temperature increase.
(3) Permafrost degradation caused by rising temperature affects the safety of expressway construction in cold regions. Based on an analysis of the settlement on the side slope of a selected section, we believe that the influence of permafrost degradation will have an increasingly significant effect on construction in cold regions.

Permafrost degradation is accompanied by soil moisture loss, land desertification, vegetation coverage reduction, groundwater pollution, and other problems, affecting the microbial permafrost, carbon cycle, and cold region ecological hydrology. Therefore, it is important to monitor the tundra in the Heihe area. However, there are many factors that influence the deformation process of permafrost. Therefore, future work should further improve deformation observation (in space and time) by combining more abundant data, and establish the interaction between climate change and permafrost degradation.

## Figures and Tables

**Table 1:** Information of data used.

No.	Acq. Date	Orbit	Frame	Mode	No.	Acq. Date	Orbit	Frame	Mode
**1**	20070610	07330	980	FBS	**11**	**20090731**	**18737**	**980**	FBS
**2**	20070726	08001	980	FBS	12	20090915	19408	980	FBS
**3**	20070910	08672	980	FBS	13	20091216	20750	980	FBD
**4**	20071211	10014	980	FBD	14	20100131	21421	980	FBD
**5**	20080126	10685	980	FBD	15	20100318	22092	980	FBD
**6**	20080427	12027	980	FBS	16	20100503	22763	980	FBS
**7**	20080612	12698	980	FBS	17	20100618	23434	980	FBS
**8**	20081213	15382	980	FBD	18	20100803	24105	980	FBS
**9**	20090128	16053	980	FBD	19	20100918	24776	980	FBS
**10**	20090615	18066	980	FBD	20	20101219	26118	980	FBD
